# Bibliometric Analysis of Electronic Cigarette Publications: 2003–2018

**DOI:** 10.3390/ijerph16030320

**Published:** 2019-01-24

**Authors:** Michael Briganti, Cristine D. Delnevo, Leanne Brown, Shirin E. Hastings, Michael B. Steinberg

**Affiliations:** 1 Rutgers Center for Tobacco Studies, 112 Paterson Street, New Brunswick, NJ 08901, USA; mb1629@sph.rutgers.edu (M.B.); delnevo@sph.rutgers.edu (C.D.D.); 2 Department of Medicine, Rutgers Robert Wood Johnson Medical School, 125 Paterson Street, Suite 2300, New Brunswick, NJ 08901, USA; leannemb@rwjms.rutgers.edu (L.B.); shirin.hastings@rwjms.rutgers.edu (S.E.H.)

**Keywords:** electronic cigarettes, e-cigarettes, electronic nicotine delivery system, bibliometric, publications, author network

## Abstract

Electronic cigarettes are at the center of a public health policy debate which leverages scientific publications. This study characterizes e-cigarette publication trends over the past 15 years via a bibliometric analysis. Scopus was searched for “electronic cigarette”, “e-cig”, “e-cigarette”, “vape”, “vaping”, “juul”, or “electronic nicotine delivery system” between 2003–2018. Data included Hirsch index, document type and frequency, and publications by institution, journal, and country. VOSviewer was used to visualize authorship network maps. A total of 4490 e-cigarette publications were identified, most (62.8%) being articles. After 2009, the annual growth rate for e-cigarette publications was the largest in 2014. The annual growth rate was nearly flat in 2017 but increased in 2018. The U.S. produced 51.6% of publications. Annual National Institutes of Health NIH funding for tobacco research mapped closely with the annual volume of e-cigarette publications. Author network analyses illustrated investigator collaborative patterns. The frequency of e-cigarette publications increased significantly in the past decade. A strong relationship of NIH funding for tobacco research and e-cigarette publications demonstrates the importance of e-cigarettes in tobacco research.

## 1. Introduction

Electronic cigarettes (e-cigarettes) are battery-powered devices that can deliver nicotine and non-nicotine solutions to the user through inhalation of a heated liquid. The first commercial e-cigarette was created in Beijing, China in 2003 [1] and, while e-cigarettes entered the United States market in 2006, e-cigarettes were initially sold over the internet or at mall kiosks. Adoption and availability increased in the early 2010s, when the market later began consolidating into traditional retail settings [2]. Consumption of e-cigarette use in the United States has steadily increased ever since [2,3,4,5].

It is well established that scientific research plays a role in the development of health policy [6,7,8]. As such, research products—in the form of publications—are important in that they are used to both inform the evidence base and contextualize public health debate [8]. Moreover, the characteristics and trends of publications can be evaluated with techniques such as bibliometric analyses. Bibliometric analyses examine the impact (using the Hirsch Index) and quantity of journal publications on a research subject over time. Common bibliometric methods include authorship analysis, citation analysis, and network visualization [9]. Specific indicators of impact and quantity include annual publication frequency, most cited articles, publications by nation, and most prolific institutions. These methods and indicators are important because they allow researchers to identify both the prominent, as well as emerging, areas of research within a field and the researchers working in those areas. This has the potential to encourage collaborative efforts among investigators.

Bibliometric analyses have been conducted on a wide range of health topics [10,11,12], and several have been conducted in the field of tobacco [13,14]. Yet only few bibliometric analyses in tobacco have been done recently [15,16], and only one bibliometric analysis specifically focused on e-cigarettes has been published to our knowledge [17]. This study found e-cigarettes are being given increased scientific attention internationally, and high scholarly productivity was noted in the US, due in large part to efforts by the Food and Drug Administration (FDA), which was granted authority over tobacco products in 2009. This single e-cigarette analysis is limited given its timeframe (only through 2014) and small sample (356 documents worldwide). The current study provides a more recent assessment of the literature, when the majority of e-cigarette publications were published, yielding a much larger sample (4490 documents). This study identifies journals, institutions, and countries disseminating the most electronic cigarette research; the frequency and amount of electronic cigarette publications over time; the most cited articles in the field; the association between National Institutes of Health (NIH) tobacco funding and publication output; keyword frequencies over time; and finally, author networks.

## 2. Materials and Methods

For this analysis, Scopus, an online database with the largest source of over 22,600 available journals across all fields of research, was used [18]. Scopus has also been used and validated in prior bibliometric analyses [17,19,20].

Search keywords included: “electronic cigarette”, “e-cig”, “e-cigarette”, “vape”, “vaping”, “juul”, and “electronic nicotine delivery system” under the “Article title, Abstract, Keywords” field, in publications between 1 January 2003 and 31 December 2018. We included documents published in all languages. A total of 4692 documents were found. Books, book series, conference proceedings, conference papers, erratum, trade publications, and undefined documents were excluded from analysis, resulting in a final sample of 4490 documents. Data on Hirsch index (h-index), document type, number of articles published annually, institutions, journals, most cited articles, top keywords, and top authors were collected. The h-index is a common bibliometric index that represents an author’s quantity and impact of publications. An author with an index of h has h papers that have attracted h citations [21]. Use of the h-index can extend beyond individual authors and can be used on journals, research topics, or geographic areas.

Document type, top institution, and top journal by frequency and percentage, amount of annual publications by frequency, and growth rate were reported. Growth rate was calculated using the following equation:

Growth Rate = [(Frequency of Current Year−Frequency of Last Year)/(Frequency of Last Year)]×100

Authorship networks were visualized using VOSviewer, which allows for the construction of bibliometric networks using citation files [22]. Citation information was imported in comma-separated value format from the Scopus search. The magnitude of the circles indicates the frequency an author was published. Circles of the same color indicate authors that are commonly grouped together. Lines connecting circles represent authors publishing together, and a thicker line represents authors publishing together more often. VOSviewer does not display names if they would lead to overlapping text; to remedy this, we manually added overlay text to display the names of authors who were not rendered in the source image. 

For the authorship network analysis, we included authors with at least 20 electronic cigarette publications. We excluded authors that did not have at least one publication with another author. There were 47 authors who met these criteria.

We obtained information on NIH funding using categorical spending data that are provided annually using the NIH Research Portfolio Online Reporting Tools (RePORT) (Bethesda, MD, USA) [23]. The funding numbers are based upon what the NIH actually funds each year, and some projects might fall under multiple categories. We mapped NIH tobacco funding to e-cigarette publications with a 5-year lag time. We began this funding analysis in 2013 because that was the first year with over 100 publications; we felt this would lead to a fairer representation of how funding could correlate with publications given a lag-time. 

We generated two keyword word clouds in TagCrowd [24]; one cloud for publications in 2014 (when the last bibliometric analysis on electronic cigarettes was conducted) and another for publications in 2018. To ensure our publication search was as inclusive as possible, we included both author-provided and index keywords in our analysis. Indexed keywords on Scopus are chosen by the publication supplier but also account for synonyms and plurals. 

## 3. Results

Of the 4490 publications from 2003–2018, the h-index was 109 as of January 2019; that is, there were 109 papers that were each cited at least 109 times. For context, the h-index of e-cigarette articles in the 2014 analysis was 27 [17]. An h-index typically grows over time. Most publications were articles (*n* = 2822, 62.8%), followed by reviews (*n* = 435, 9.7%), and notes (*n* = 396, 8.8%). Letters (*n* = 358, 8.0%), editorials (*n* = 225, 5.0%), articles in press (*n* = 161, 3.6%), and short surveys (*n* = 93, 2.1%) made up the remaining electronic cigarette publications.

**Figure 1:**
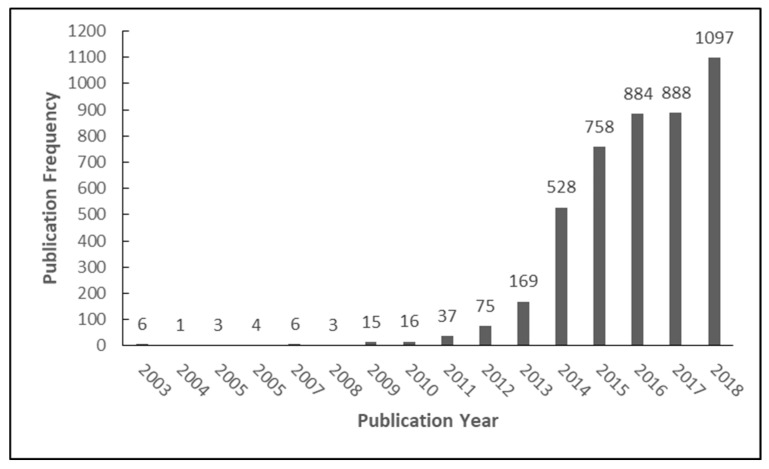
Annual electronic cigarette publication frequency from 2003 to 2018.

The frequency of e-cigarette publications by year is seen in Figure 1. Prior to 2009, annual publication frequency was in the single digits, making the annual growth rate sporadic and inconsistent. Between 2010 and 2014, the number of annual publications at least doubled each year. The largest growth in publications since 2008 was between 2013 and 2014, from 169 to 528 publications, an annual growth rate of 212%. There was very little growth in 2017 when compared to 2016, but the amount of publications in 2018 increased by nearly 24% from the prior year.

The most prolific institutions with electronic cigarette publications from 2003–2018 included the University of California, San Francisco (145 publications; 3.0%), Johns Hopkins Bloomberg School of Public Health (92; 2.0%), and the University of North Carolina at Chapel Hill (91; 2.0%). The Virginia Commonwealth University, University of Southern California, Roswell Park Cancer Institute, King’s College in London, Centers for Disease Control and Prevention, and the Food and Drug Administration—Center for Tobacco Products all had fewer than 2% of publications each but were the next most prolific institutions. Of the top 10 most published institutions, nine were based in the United States and one was based in the United Kingdom.

After excluding those articles with “undefined” countries or territories, the United States produced 1666 publications (51.9%), the United Kingdom had 443 publications (13.8%), followed by Australia with 156 publications (4.9%). Italy, Canada, Germany, France, Switzerland, Greece, and China were also included in the top 10 countries publishing e-cigarette papers.

The journals containing the most electronic cigarette publications included: *Nicotine and Tobacco Research* (212; 4.7%), *Tobacco Control* (197; 4.4%), and *Addictive Behaviors* (149; 3.3%). *The International Journal of Environmental Research and Public Health*, *Addiction*, *Drug and Alcohol Dependence*, *BMJ Online*, *Preventive Medicine*, *American Journal of Preventive Medicine*, and *PLOS One* were the remaining top 10 journals.

The most cited electronic cigarette publications are indicated in Table 1. Of note, the top 10 most cited publications include five articles and five reviews. 

**Figure 2:**
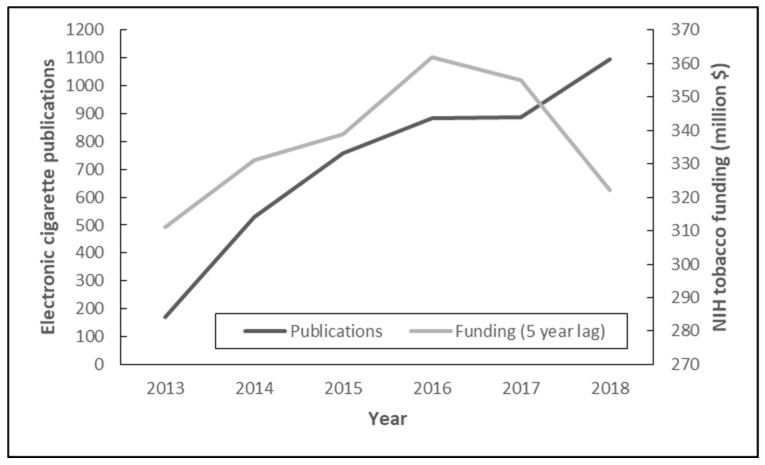
National Institutes of Health (NIH) tobacco funding with a 5-year lag time and electronic cigarette publications from 2013 to 2018.

Figure 2 shows NIH tobacco research funding with a 5-year delay and the associated amount of e-cigarette publications. Findings indicate a rising number of publications from 2013 and 2016 that followed an increase in tobacco research funding from 2008 to 2011. Conversely, there was about the same number of publications in 2017 compared to 2016, following decreased NIH tobacco research funding in 2012. However, there was a large drop in tobacco funding in 2013 but a large growth of publications in 2018. 

**Figure 3:**
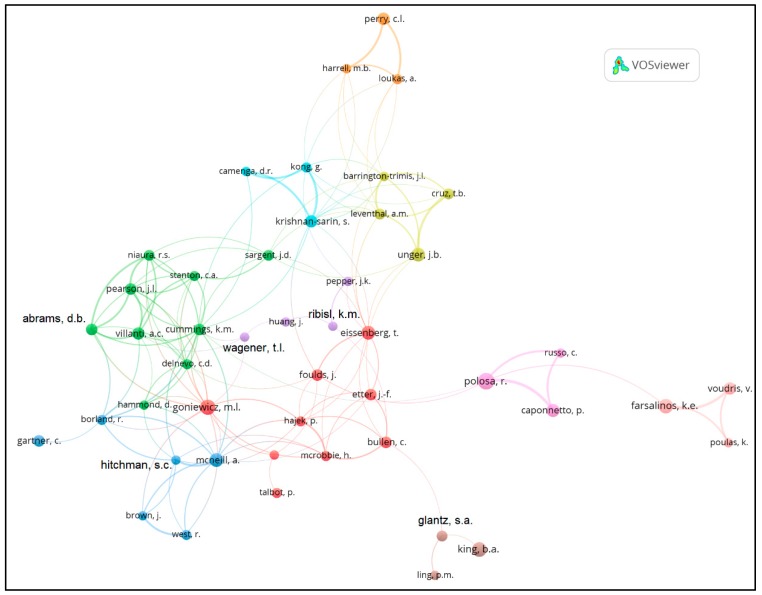
Network map of top authors in electronic cigarettes from 2003 to 2018. Authors were required to have at least 20 publications for inclusion. Colors indicate typical co-authors.

The network of authorship in the field of electronic cigarettes is shown in Figure 3. The figure shows collaboration in the field of electronic cigarettes within and between clusters of investigators. There are clear authorship clusters. For instance, Goniewicz links to McNeill and Abrams. Beyond the large collaborative cluster, there is a smaller cluster which includes Perry, and then several other authors isolated from the most prolific electronic cigarette authors. 

**Figure 4:**
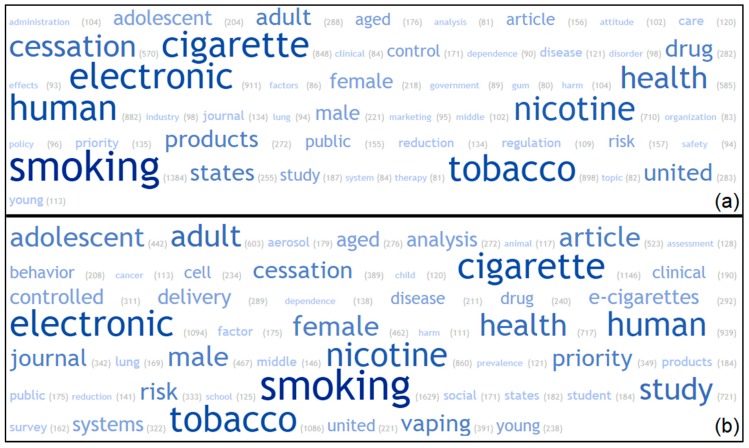
(**a**) Top 50 author-provided and indexed keywords in electronic cigarette publications in 2014. (**b**) Top 50 author-provided and indexed keywords in electronic cigarette publications in 2018. Bold text indicates the proportion of keyword mentions, and the number in parentheses shows the frequency of use.

The top 50 indexed and author-provided keywords are shown in Figure 4. The top image displays keywords from 2014 publications, and the bottom image displays keywords from 2018 publications. Keywords are displayed in alphabetical order for increased readability and comparison. Overtime, the main search keywords remain relatively consistent. “United” and “states” are found in both images, making the United States the only country to appear in the top 50 keywords. 

In 2018, some keywords increased in the proportionality of usage. These include “adolescent”, “behavior”, “clinical”, “lung”, and “vaping”. In 2018, new keywords emerged in the top 50, including “cancer”, “child”, “school”, and “student”. Other keywords like “cessation” and “drug” had a decreased proportion of usage in 2018.

## 4. Discussion

This bibliometric analysis demonstrates that the amount of publications in the field of electronic cigarette research increased notably as e-cigarettes grew in popularity, as well as the health concerns surrounding their use as well as their potential as a harm reduction product. Although e-cigarette research has grown quickly, there was stagnation in 2017, followed by another rise in 2018.

In considering influences on the growth pattern of e-cigarette publications, the funding of tobacco research is certainly a contributing factor. The trajectory of e-cigarette publications, with a 5-year time lag, maps very closely onto NIH tobacco research funding, except in 2018. Funding increased steeply between 2008 and 2010, then slowed in 2011 and flattened in 2012. The corresponding e-cigarette publication curve was very steep from 2013 to 2015 and flattened in 2016 and 2017. This 5-year lag could align with the time frame of obtaining funding, conducting a study, and preparing and publishing the results. The fact that tobacco research funding, not specific e-cigarette research funding, mapped so closely onto e-cigarette publications, illustrates that electronic cigarette research was a rapidly growing area within the tobacco field. Funding initiatives should be sure not to neglect emerging health issues, such as e-cigarettes, to drive scholarly output that enhances the success of current tobacco control efforts. The inverse relationship between funding in 2013 and publications in 2018 raises questions, but the link between funding and scholarly output should be studied in greater detail in future research.

The network map of authors provides interesting insight as to the e-cigarette scholarship landscape. These collaborative author groupings or clusters tend to have a distinct theme and/or are geographically grouped. For examine, the cluster in yellow (Leventhal, Barrington-Trimis, Unger, and Cruz) is based at the University of Southern California and their research focuses primarily on youth e-cigarette use and concerns regarding gateway issues. The cluster in green (which includes but is not limited to Abrams, Villanti, Pearson, and Cummings) is a diverse group whose work focuses on population-level policy and harm-reduction issues. The cluster in red (which includes but is not limited to Goniewicz and Eisenberg) focuses on e-cigarette products and features, such as exposure and constituents. The cluster in pink (e.g., Caponetto, Polosa, Farsalinos) are EU researchers who were some of the early investigators in harm reduction. Of note, this cluster as well as the orange (Perry et al.,) and brown cluster (Glantz) are somewhat isolated. Through this type of novel information, one can see how the spectrum of e-cigarette research activity interacts as a function of the themes and authors themselves. This approach could help the research community to better explore cross-disciplinary and cross-institutional, and perhaps most importantly, cross-“belief-system” efforts.

Interestingly, four of the top 10 articles with the most citations were from journals that did not make the list of journals with the highest volume of e-cigarette manuscripts. Reviews made up under 10% of all electronic cigarette publications but made up half of the top 10 most cited electronic cigarette publications. These facts illustrate the difference between publication volume (quantity) and impact. In recent years, e-cigarette use has become a more prevalent public health problem, and thus, articles regarding their use have appeared in more high-impact sources, including the *Journal of the American Medical Association* and the *New England Journal of Medicine*. There may be so few articles in these journals to date that they will not show up in bibliometric analyses yet and, thus, are harder to analyze. However, their impact on policy decisions could be very important.

Most research in the e-cigarette field is conducted by United States researchers and institutions. As seen in Figure 4, in both 2014 and 2018, the United States is the only country that appears in top 50 publication keywords. Previous bibliometric analyses on e-cig publications had similar results [17], as well as a recent bibliometric analysis of global nicotine replacement therapy publications [35]. The keyword analysis provides information on the direction of e-cigarette research. Publications in 2018 had emerging keywords dedicated to adolescents, children, and schools compared to prior years, possibly indicating increased concerns about e-cigarette usage among youth. A decreased use of keywords like “cessation” potentially indicate that there is less interest in that area of research, and increased interest elsewhere. 

## 5. Conclusions

This study expands upon previous efforts to analyze the literature surrounding electronic cigarettes, using a more inclusive search method. One limitation of this study is that institutions and nations are given the “credit” solely based upon the first author’s affiliation. This might make it appear as though there is less collaboration in the field than there is in reality. Despite this, we conducted a thorough search using Scopus, used more comprehensive and up to date e-cigarette terminology, and included more recent publications. The inclusion of publications after 2014 allowed us to capture 2018’s large growth in publications after 2017’s stagnation. This study provides the first publication keyword analysis in the field of electronic cigarettes and might provide insight to tobacco researchers on emerging areas of research within the field. This study also provides the first author network analysis for e-cigarette research, and authors might be able to use this information to identify other groups to collaborate with in the future.

## Figures and Tables

**Table 1:** Top 10 electronic cigarette articles from 2003 to 2018.

Rank	Title	Year	Journal	Citations	Type	Reference
1	Heart Disease and Stroke Statistics—2017 Update: A Report From The American Heart Association	2017	*Circulation*	1928	Review	[25]
2	2016 European Guidelines on cardiovascular disease prevention in clinical practice	2016	*European Heart Journal*	1166	Review	[26]
3	Levels of selected carcinogens and toxicants in vapour from electronic cigarettes	2014	*Tobacco Control*	629	Article	[27]
4	Electronic cigarettes for smoking cessation: A randomised controlled trial	2013	*The Lancet*	609	Article	[28]
5	E-cigarettes: a scientific review	2014	*Circulation*	536	Review	[29]
6	Electronic cigarette: Users profile, utilization, satisfaction and perceived efficacy	2011	*Addiction*	411	Article	[30]
7	EffiCiency and Safety of an eLectronic cigAreTte (ECLAT) as Tobacco Cigarettes Substitute: A Prospective 12-Month Randomized Control Design Study	2013	*PLOS One*	400	Article	[31]
8	Electronic Nicotine Delivery Systems: International Tobacco Control Four-Country Survey	2013	*American Journal of Preventive Medicine*	355	Review	[32]
9	Effect of an electronic nicotine delivery device (e cigarette) on desire to smoke and withdrawal, user preferences and nicotine delivery: randomised cross-over trial	2010	*Tobacco Control*	298	Article	[33]
10	e-Cigarette Awareness, Use, and Harm Perceptions in US Adults	2012	*American Journal of Public Health*	319	Review	[34]
